# Chinese English as a Foreign Language Learners’ Individual Differences and Their Willingness to Communicate

**DOI:** 10.3389/fpsyg.2022.883664

**Published:** 2022-06-29

**Authors:** Ling Cheng, Jieping Xu

**Affiliations:** ^1^School of Humanities and International Education and Exchange, Anhui University of Chinese Medicine, Hefei, China; ^2^School of Liberal Arts, Shinawatra University, Bang Toei, Thailand; ^3^School of Foreign Languages, Chaohu University, Chaohu, China

**Keywords:** communication skills, willingness to communicate, individual differences, interaction, verbal or non-verbal communication, contextual factors

## Abstract

One of the most significant issues in the success of language students is communication skills. Due to the high importance of the willingness to communicate in foreign language learning, many researchers are looking for effective factors in raising the level of communication among language learners. Reviewing the literature, the researchers explored the role of language learners’ gender, major, age, and proficiency level in their willingness to communicate. To gather the necessary data, the researcher administered a willingness to communicate questionnaire among 860 Chinese english as a foreign language (EFL) students. The results of data analyses demonstrated that gender, major, and age played a significant role in determining language learners’ willingness to communicate. However, the findings showed that learners’ willingness to communicate could be different in all proficiency levels. The results showed that taking into account factors such as students’ gender, background, and age directly helps to improve the willingness to communicate among language learners. Finally, solutions to improve the willingness to communicate are provided.

## Introduction

Human beings are social creators that making regular connections between members of society is one of the obvious needs. When people communicate, they do not take expressions out of their mouths for any specific purposes, but aim to communicate with and address others (Aubrey, 2011). We are not just trying to voice our thoughts, but our goal is to influence our audience or audiences. By talking, we would like the other person to comprehend what we mean, and he listens to us to understand what we mean (Kang, 2014; Derakhshan et al., 2022). Communication is an important and central factor in our lives that, if done correctly, can prevent stressful situations and lead to the solution of many problems in life. Effective communication with others reduces the severity of stress. The way we relate to others and their reactions is rooted in self-esteem (Lee, 2018). Communication is the process of transmitting a message from the sender of the message to the recipient of the message, provided that semantic similarity occurs. We interact with others in different ways every day and exchange our thoughts, feelings, and desires (Boudreau et al., 2018; Derakhshan et al., 2019).

The most important way to achieve positive results is to satisfy our needs and fulfill our desires in communication (Cao, 2011; Elahi Shirvan et al., 2019). Today’s world is the world of communication; we spend most of our lives in connection with others or think about it. Although communication may seem simple, it requires the use of tips that will not be effective if not used properly and in a timely manner (Yashima, 2002; Fathi et al., 2021). If we can communicate more effectively and successfully, we will achieve positive results in life. It is through communication with others that we can achieve our goals and aspirations and achieve significant progress and success in our daily plans and activities. In fact, our communication skills indicate our ability and self-confidence and increase our value and respect for others (Khajavy et al., 2016; Yashima et al., 2018; Mellati et al., 2022). These skills have a direct impact on our progress, responsibility, and career path, and on the amount of support and help we receive from others and even enable us to persuade others to persuade them to do what we want them to do. Communication includes verbal and non-verbal communication (Bernales, 2016; Greenier et al., 2021).

Students’ communication, whether in the school or outside the school environments, is not outside this framework, meaning that students’ interactions, verbal or non-verbal communication, affects their performance (Boudreau et al., 2018; Alavi et al., 2021). Communication in foreign language classes is one of the most controversial subjects in the field of language teaching, which is not only a challenge for language learners; rather, because of its great importance, it always attracts the attention of researchers in this field. Research in this area is often done to find a model that meets the needs of the learners (Cao and Philp, 2006; Dewaele and Dewaele, 2018; Derakhshan, 2021). Unwillingness to communicate in another language, especially in foreign language contexts, is a problem that exists in most learning situations (Di Silvio et al., 2016; Khajavy et al., 2017; Fathi and Derakhshan, 2019; Pishghadam et al., 2020). This means that learners refuse to communicate or consider it insignificant (Liu and Jackson, 2008). One of the main reasons learners are reluctant to communicate is the fact that they are concerned about their respect and credibility, and since there is always the possibility of a negative reaction from other students in the class to what they say, they are afraid that their reputation will be tarnished (Mellati et al., 2013). Even after several years, they still have no desire to express themselves. This reluctance may lead to poor communication skills that are an integral part of their speaking skills (Peng, 2012; Reid and Trofimovich, 2018; Pishghadam et al., 2021).

One of the important factors in learning a second or foreign language is learning motivation. The stronger the motivation to learn, the more diligent learners go through the grueling path of language learning and enjoy learning (Denies et al., 2015; Mellati and Khademi, 2015; Tavakoli and Zarrinabadi, 2016). In recent decades, many english as a foreign language (EFL) researchers have emphasized the vital role of motivation in the language learning process. According to Lu and Hsu (2008), motivation provides learners with the motivation to begin and continue learning. However, in the present age, many researchers believe that motivation is just a general term to describe success or failure in language learning, and that motivation to learn a second or foreign language is affected by various variables (Nematizadeh and Wood, 2019; Wang Y. L. et al., 2021).

Willingness to Communicate (WTC) is one of the important motivational variables that emphasizes the need to communicate through talking (Denies et al., 2015; Di Silvio et al., 2016; Derakhshan et al., 2020). It helps learners to use their language knowledge in different situations and subconsciously understand what to say, when, how, and to whom. However, language learners vary in their WTC; for example, some learners express their ideas and opinions freely and participate in class discussions (Jamalifar and Salehi, 2020), but some prefer to remain silent and not to participate in class discussions (Joe et al., 2017). In fact, some learners are looking for adequate opportunities to speak and use a second or foreign language in the classroom, while another group of learners is reluctant to speak or even refuse to speak (MacIntyre and Legatto, 2011).

The ability to communicate effectively is one of the most important fundamentals in the language teaching development (Peng, 2013; Derakhshan et al., 2020). This ability is reinforced in the conversational class within language learners. In this way, the instructor encourages the learner to speak in class and participate in the discussion according to the teaching method and the way of feedback (Peng and Woodrow, 2010). However, there are also problems for language learners who react differently to it, either because of individual and personality differences or because of the conditions prevailing in foreign language conversation classes. Students, like other sections of society, may have deficiencies in social relationships and assertive behaviors; but because they will have more relationships in the future, they need more communication skills (Mellati et al., 2015b; Bernales, 2016; Vongsila and Reinders, 2016). A high percentage of students seem to have difficulty expressing appropriate social responses and methods of social and professional communication with others (Yashima et al., 2018). Many of them seem to have grown up in environments that did not have the opportunity to learn social skills. Although there is no comprehensive study in Chinese society that has examined these problems; the evidence suggests that many Chinese students have difficulty coping with situations, expressing emotions properly, being afraid to speak in public, and feeling shy and embarrassed, and are unable to exercise their rights. In addition, several studies found that many crucial factors such as gender, age, proficiency level, context, and culture affect learners’ WTC; therefore, the current study inspected Chinese foreign language learners’ WTC and focused on different aspects of WTC in the Chinese context.

## Review of Literature

In foreign language conversation classes, the goal is to persuade language learners to speak in public and participate in classroom activities, to develop language skills and to increase their desire to communicate.

## Theoretical Framework

In this research, Bandura’s social learning theory has been used as a theoretical framework and central theory. Social learning theory is another interpretation of behaviorism (Zhong, 2013). In this view, students learn from others not only through reward and conditioning but also through observation and imitation. Bandura’s emphasis on the importance of cognitive factors in the concept of observational learning is described. According to him, learning is not limited to the conditioning of behavior or trial and error (Joe et al., 2017). Learning is often shaped by observing the behavior of others such as parents, peers, teachers, and even movie and cartoon scenes. Learning complex behaviors such as driving, swimming, and surgery cannot be justified if learning is limited to trial and error (Wood, 2016).

To avoid the dangers of trial and error, these behaviors are learned by observing, reading storybooks, or even listening to instructions. He calls this type of learning substitution learning in which the learner symbolically puts himself in the place of the model and at the same time learns his experience (Mellati and Khademi, 2014; Bernales, 2016; Tavakoli and Zarrinabadi, 2016). Like Skinner, Bandura emphasizes that human behavior and personality are acquired; That is, it is formed by learning. However, he does not agree with the classical behaviorists about the learning process. In his view, the processes that govern animal learning do not necessarily apply to humans. Human being is social and his behavior should be examined in the light of social relations. Man has vast cognitive capacities and can think about the relationship between behavior and its consequences and predict and evaluate them. Bandura believes that observational learning is a type of cognitive learning that includes the four processes of attention, maintenance (retention), production (motor reconstruction), and reinforcement (motivation) (Peng and Woodrow, 2010; Reid and Trofimovich, 2018).

The first dimension, that is, language abilities are strengthened over time and according to the students’ reading rate (Nematizadeh and Wood, 2019). In this way, students who speak and are more fluent in a foreign language will have more and more effective tools for communication. The second dimension, or the tendency to communicate, is influenced by other variables in addition to individual effort (Mellati et al., 2015a; Di Silvio et al., 2016; Fathi and Derakhshan, 2019). One of the elements that can reduce a learner’s performance, especially in language conversation classes, is shyness and unwillingness to communicate. Strategies for communication are a mutual effort between two audiences to better understand each other’s message (Peng, 2013; Mellati and Khademi, 2018). Communication is considered successful when the parties feel responsible for understanding the other’s purpose and understanding their own purpose, and when they realize that this is not achieved, they seek compensation and change the wording, using simple and more understandable patterns, and the like. They try to understand what they are saying (Jamalifar and Salehi, 2020).

## Willingness to Communicate

In today’s vast world, the desire to communicate is the major objective of language learning. The notion of WTC was first introduced in 1976 by Burgoon (1976) under the title “unwillingness to communicate.” Then, Baer and McCroskey (1985) introduced this concept in second or foreign language teaching and learning. The WTC in a second or foreign language means taking advantage of opportunities to communicate with others in another language. The importance of the desire to communicate stems from the reality that the main purpose of language teaching is to prepare learners to express their intended meanings and concepts in the target language, and in this case, the unwillingness to communicate reduces the success rate in learning a foreign language.

In addition, many linguistic theories and hypotheses, such as the Interaction Hypothesis, the Socio-cultural Hypothesis, and the Output Hypothesis emphasize the need for interaction and communication in second language learning (Mystkowska-Wiertelak and Pawlak, 2014). The tendency to communicate as an important motivating factor is also related to the method of communication teaching and group language learning. In these methods, collaboration between learners and the use of a second or foreign language to communicate with others is very important. The desire to communicate was first introduced by Baer and McCroskey (1985) for learning the mother tongue; However, Cetinkaya (2005) observed that the tendency to communicate in a second or foreign language is not a fixed characteristic and can change under different factors. Then, by presenting an exploratory model, they tried to show the factors influencing it. In general, the exploratory model (Cao and Philp, 2006; Aubrey, 2011) has two variables: Transient variables that include environmental factors such as classmates, teacher, classroom atmosphere, and persistent variables such as anxiety and motivation.

Since the introduction of this model, researchers have tried to examine the relationship between the desire to communicate with other environmental and psychological factors, to help teachers to increase or decrease the level of such variables, increase the level of desire to communicate among their learners (Denies et al., 2015), and ultimately help develop their second or foreign language. Extensive and diverse research has been conducted with a similar approach in the field of English language teaching, which shows the close relationship between shyness and inversion with the tendency to speak. Perceptual social self-efficacy plays a major function in the severity of learners’ anxiety and is a vital factor in their overall performance and effective learning. Also, high introversion leads to a weak sense of self-confidence, increased anxiety and, consequently, decreased performance of language learners in foreign language classes (Dewaele and Dewaele, 2018; Mellati and Khademi, 2020; Wang and Guan, 2020).

Other factors, such as having a high level of emotional intelligence, which has a positive effect on the motivation for progress, reduce the anxiety of the language class (Jamalifar and Salehi, 2020). The scope of this research is not limited to language learners; but also the role of the teacher as a determining factor in the process of learning-teaching review and motivational task-orientation makes him more important and vital tasks than other tasks (Kang, 2014). Another factor that has been studied on the effect of the learning-teaching process is the empathy between the teacher and the learner, which improves this process by providing a favorable atmosphere (Khajavy et al., 2016). In the field of language teaching, the effectiveness of foreign language teaching is measured by the rate of student participation; but learners differ for the language they use inside and outside the classroom (Khajavy et al., 2017). When given the opportunity to use a second language, some choose to speak loudly and take every opportunity to communicate, although they may not speak fluently. Others speak only when they are addressed, which is likely to prevent them from speaking at that time as well (Lee, 2018; Mellati et al., 2018).

Many studies explored WTC from different aspects: Dewaele (2019) explored the effect of emotion on WTC and found that anxiety is a negative predictor of WTC while pleasure and language use frequency were positive predictors of WTC. In another study, Wang H. et al. (2021) stated that many factors such as age, gender, and personality traits are contributed to learners’ WTC. Khany and Nejad (2017) explored the relationship between WTC and personality traits. They found that openness to experience and extraversions were the main predictors of WTC. Fernández-García and Fonseca-Mora (2022) inspected the relationship between language proficiency and emotional understanding to WTC. They found that while there is no difference between learners in different proficiency levels and their tendency to communicate, emotional understanding had a significant impact on their WTC. While they found no significant difference on WTC across proficiency level, Zare et al. (2020) investigated learners’ WTC across proficiency levels and found a statistically significant difference. Lee et al. (2019) investigated the impact of geographical context on language learners’ WTC. They compared Korean and Taiwanese language learners’ WTC and found that there was no significant differences between them in WTC. However, they found that while Korean language learners had greater WTC outside the classroom than Taiwanese learners, the Taiwanese outperformed on WTC in technology-enhanced contexts than did the Koreans. In contrast, Yashima et al. (2018) investigated the impact of context and individual individuality on learners’ WTC. The results of their analyses showed that learners’ characteristics such as language proficiency, personality, and contextual factors influence their WTC.

In another study, Pawlak et al. (2016) examined the nature of classroom and its effect on learners’ WTC. Similar to Yashima et al. (2018), they found that contextual and individual factors influenced learners’ WTC. Lan et al. (2021) focused on shyness as one of the main predictors of WTC among language learners and found that numerous factors should be considered in the determination of language learners’ WTC. Zhang et al. (2018) stated that while numerous studies have been conducted on language learners’ WTC, there are still many questions regarding the impact of age, gender, proficiency level, emotional, and contextual factors on WTC. Therefore, the present study investigated Chinese language learners’ WTC in terms of gender, age, and English proficiency levels.

## Research Questions

To solve the above-mentioned problem, the present study was conducted to investigate Chinese EFL learners’ WTC and answer the raised questions:

RQ1: Are there any statistically significant differences in Chinese EFL learners’ WTC in terms of their gender?

RQ2: Are there any statistically significant differences in Chinese EFL learners’ WTC in terms of their major?

RQ3: Are there any statistically significant differences in Chinese EFL learners’ WTC in terms of their age?

RQ4: Are there any statistically significant differences in Chinese EFL learners’ WTC in terms of their English level?

## Materials and Methods

### Participants

A sample of 860 Chinese EFL students (valid cases) was collected with a convenience sampling strategy in China from different universities and colleges across 10 provinces, one Autonomous Region, and two municipalities. The sample comprises 230 males (26.7%) and 630 females (73.3%), with their ages ranging from 16 to 22, 483 (56.2%) of them majoring in liberal arts and 377 (43.8%) in sciences. The EFL learning level of our participants ranges from undergraduates to postgraduates; the English level of the participants were based on the universities’ placement tests. Informed consent was given to all participants. The detailed demographic information is presented in Table 1.

**TABLE 1:** Demographic information of the language learners.

Information category	*N*	*%*
**Gender**		
Male	230	26.7
Female	630	73.3
Total	860	100
**Age**		
16–18	142	16.5
19–21	597	69.4
22–24	121	14.1
Total (Valid)	860	100
Total	860	100
**Majors**		
Liberal arts	483	56.2
sciences	377	43.8
Total	860	100
**English levels**		
primary	255	30
intermediate	365	42
high	240	28
Total (Valid)	860	100
Total	860	100
**Geographic distribution (Province)**		
Anhui	258	30
Inner Mongolia	174	20
Guizhou	152	18
Liaoning	74	9
Guangdong	64	7
Chongqing	54	6
Henan	48	6
Others	36	4
Total	860	100

### Willingness to Communicate Questionnaire

The WTC questionnaire by MacIntyre et al. (2001) was administered to measure the participants’ degrees of WTC. The questionnaire is composed of 54 items that measure the respondent’s degree of WTC across a range of different communicative situations inside and outside of language classrooms. The questionnaire uses a scale of 1–5 for degrees of WTC, where 1 stands for “almost never willing” and 5 for “almost always willing.” The questionnaire was used to enhance the participants’ understanding of the WTC construct. In addition, the responses provided information on the participants’ degrees of WTC. To avoid misunderstanding, the researchers translated the questionnaire into China. At first, the questionnaire translated by two bilingual translators. Then, to ensure the accuracy of the translation, the researchers back-translated the initial translation independently. Then, three experts in the field checked the prefinal version of the questionnaire. The questionnaire finally was piloted by thirty participant of the same study. The results of Cronbach Alpha analysis show the reliability index of 0.78 (α = 0.78).

### Data Collection Procedure

The questionnaire containing 27 questions was designed by Liu et al. (2021), the permission of a representational study was asked in advance in China. After a Chinese version of the translation by the authors, it was posted on Wenjuanxing, a Chinese online website for designing and distributing the questionnaire, and was later shared through WeChat, one of the most popular Chinese social media platforms. Participants used their cell phones to scan the questionnaire QR code and completed it under detailed instructions. Prior to accessing the scales, participants were presented with a consent form explaining the general idea of the present study, the estimated time of completion, their right to withdraw from the study at any moment, and the risk for their participation. More importantly, participants were clearly informed that their anonymity would be ensured, and their collected data would be only employed for research purposes under the anonymous premise. Finally, a declaration in participants’ tone was presented with a dual-option (“Yes” or “No”) asking their willingness to participate. Participants who answered “No” were automatically redirected to the end of the questionnaire. To avoid misunderstanding for low English reading ability and a lack of patience for longer reading time, we used the Chinese scales which may result in the enhancement of the validity and reliability notions of the questionnaire. Before accessing each scale, participants were presented with detailed instructions on what the following scale is measuring and how to respond to each item. Although there was no time limit to completing the scale, participants took 497 s to submit their response on average. The QR code of the questionnaire was shared on WeChat with a large group of EFL students throughout the entire country. The data collection process took more than 1 month, from December 2021 to January 2022. Eventually, 860 EFL college learners participated in this study, all responses as our sample.

## Results

RQ1: Are there any statistically significant differences in Chinese EFL learners’ WTC in terms of their gender?

To answer the first research question, the researcher ran an independent-samples *T*-Test. The results of this analysis are presented in Tables 2, 3.

**TABLE 2:** Descriptive analysis for gender.

Gender	*N*	Mean	Std. deviation	Std. error mean
Male	230	88.29	28.152	1.856
Female	630	94.53	24.393	0.972

**TABLE 3:** The results of willingness to communicate (WTC) differences in terms of gender.

	Levene’s test for equality of variances		*T*-test for equality of means						
		
	*F*	Sig.	*t*	df	Sig. (2-tailed)	Mean difference	Std. error difference	95% confidence interval of the difference	
								
								Lower	Upper
Equal variances assumed	7.910	0.005	−3.183	858	0.002	−6.242	1.961	−10.090	−2.393
Equal variances not assumed			−2.979	361.846	0.003	−6.242	2.095	−10.362	−2.121

The results of Table 2 show that female learners had more willingness to communicate than their male peers (Males: M = 88.29, SD = 28.152, Females: M = 94.53, SD = 24.393).

The researcher conducted an independent-samples *t*-test to compare the WTC scores for males and females language learners. There were significant differences in scores for male learners and females *t*_(361.846)_ = –2.979, *p* = 0.003, two-tailed. The extent of the differences in the mean scores were very small (eta squared = 0.01).

RQ2: Are there any statistically significant differences in EFL Chinese learners’ WTC in terms of their major?

To answer the second research of the study, and the differences between two groups, the researcher conducted an independent-samples T-Test. The results of this analysis are presented in Tables 4, 5.

**TABLE 4:** Descriptive analysis for major.

Major	*N*	Mean	Std. deviation	Std. error mean
Liberal arts	483	95.29	25.350	1.153
Sciences	377	89.75	25.583	1.318

**TABLE 5:** The results of willingness to communicate (WTC) differences in terms of major.

	Levene’s test for equality of variances		*T*-test for equality of means						
		
	*F*	Sig.	*t*	df	Sig. (2-tailed)	Mean difference	Std. error difference	95% confidence interval of the difference	
								
								Lower	Upper
Equal variances assumed	0.054	0.816	3.164	858	0.002	5.535	1.749	2.102	8.968
Equal variances not assumed			3.161	804.532	0.002	5.535	1.751	2.098	8.972

The results of Table 4 show that those who studied liberal arts had more willingness to communicate than their peers who studies sciences (Liberal arts: M = 95.29, SD = 25.350, Sciences: M = 89.75, SD = 25.583).

The researcher conducted an independent-samples t-test to compare the WTC scores for Liberal arts and Sciences learners. There were significant differences in the questionnaire scores for Liberal arts learners and Sciences *t*_(858)_ = 3.164, *p* = 0.002, two-tailed. The amount of these differences in the means scores was very small (eta squared = 0.01).

RQ3: Are there any statistically significant differences in EFL Chinese learners’ WTC in terms of their age?

To answer the third research question and find the differences among three groups, the researcher conducted ANOVA. The results of these analyses are presented in Tables 6–8.

**TABLE 6:** Descriptive analysis for age.

Age	*N*	Mean	Std. deviation	Std. error	95% confidence interval for mean		Minimum	Maximum
					
					Lower bound	Upper bound		
16–18	142	89.35	26.518	2.225	84.95	93.74	28	140
19–21	597	95.06	24.710	1.011	93.08	97.05	28	140
22–24	121	86.11	27.248	2.477	81.20	91.01	28	140
Total	860	92.86	25.586	0.872	91.15	94.57	28	140

**TABLE 7:** The results of ANOVA for age.

Total score					

	Sum of squares	df	Mean square	*F*	Sig.
Between groups	10170.699	2	5085.350	7.893	0.000
Within groups	552149.276	857	644.282		
Total	562319.976	859			

**TABLE 8:** The results of Scheffe test of multiple comparisons for age.

(I) Age		(J) Age		Mean difference (I–J)	Std. error	Sig.	95% confidence interval	
							
							Lower bound	Upper bound
Dimension2	16–18	dimension3	19–21	–5.719	2.370	0.055	–11.53	0.09
			22–24	3.238	3.140	0.588	–4.46	10.94
	19–21	dimension3	16–18	5.719	2.370	0.055	–0.09	11.53
			22–24	8.956*	2.531	0.002	2.75	15.16
	22–24	dimension3	16–18	–3.238	3.140	0.588	–10.94	4.46
			19–21	−8.956*	2.531	0.002	–15.16	–2.75

*The mean difference is significant at the 0.05 level.

The results of Table 6 show that those learners who were between 19-21 had more willingness to communicate than their peers who were in less or more ages (16-18: M = 89.35, SD = 26.518, 19-21: M = 95.06, SD = 24.710, M = 86.11, SD = 27.248).

The researcher conducted a one-way between-groups analysis of variance to discover the impact of age on WTC as measured by the WTC questionnaire. Participants were divided into three groups according to their age (G1: 16–18; G2: 19–21; and G3: 22–24). There were statistically significant differences at the *p* < 0.05 level in questionnaire scores for the three groups: *F*_(2,857)_ = 7.893, *p* = 0.000. Despite the statistical difference, the actual difference in mean scores between the groups was quite small. The effect size of the study calculated using eta squared was 0.01.

*Post-hoc* comparisons employing Scheffe test designated that the questionnaire mean score for 19–21 (M = 95.06, SD = 24.710) were significantly different from 22–24 (M = 86.11, SD = 27.248).

RQ4: Are there any statistically significant differences in EFL Chinese learners’ WTC in terms of their English level?

To answer the fourth research question and find the differences among the three groups, the researcher conducted ANOVA. The results of these analyses are presented in Tables 9, 10.

**TABLE 9:** Descriptive analysis for English level.

	*N*	Mean	Std. deviation	Std. error	95% confidence interval for mean		Minimum	Maximum
					
					Lower bound	Upper bound		
Primary	255	90.15	27.548	1.725	86.76	93.55	28	140
Intermediate	365	94.59	23.084	1.208	92.21	96.97	28	140
Advanced	240	93.10	26.874	1.735	89.69	96.52	28	140
Total	860	92.86	25.586	0.872	91.15	94.57	28	140

**TABLE 10:** The results of ANOVA for English level.

	Sum of squares	df	Mean square	*F*	Sig.
Between groups	2974.188	2	1487.094	2.278	0.103
Within groups	559345.787	857	652.679		
Total	562319.976	859			

The numbers of Table 9 show that there was no significant differences in learners’ willingness to communicate in different English levels (Primary: M = 90.15, SD = 27.548, Intermediate: M = 94.59, SD = 23.084, M = 93.10, SD = 25.586).

The researcher conducted a one-way between-groups analysis of variance was to discover the impact of learners’ proficiency level on WTC as measured by the WTC questionnaire. Participants were divided into three groups according to their English level (G1: Primary; G2: Intermediate; and G3: Advanced). There was no statistically significant difference at the *p* < 0.05 level in questionnaire scores for the three groups: *F*_(2,857)_ = 2.278, *p* = 0.103.

## Discussion

Numerous factors affect the way language learners perform and their WTC. The present study, considering the importance of this issue, sought to solve the problems of students in this field to some extent by examining factors such as gender, age, and proficiency level. The particular results of this study discovered that there were statistically significant differences between male and female language learners. The results showed that female language learners are more eager to participate in communication activities in language classrooms. The results were in consistent with what Aubrey (2011) and Khany and Nejad (2017) argued. They stated that students’ reluctance to communicate in a foreign language, which means a tendency to avoid oral conversation, is one of the main problems for language teachers. Therefore, if the source of this reluctance is identified, language teachers can make better decisions about helping learn language learners who seem reluctant to communicate and usually do not engage in interactions. However, the extent of this reluctance is not the same in different genders and may be due to different reasons. They believed that personality traits such as gender could affect language learners’ WTC.

The results of this study also demonstrated that language learners’ background knowledge take parts in a key role in their WTC. The results showed that there was a significant difference between learners with different majors and language backgrounds. One of the important issues in the sociology of education is the discussion of academic success and educational experiences based on excellence, knowledge reserves and learning experiences according to the economic and cultural situation of families and students. Similar to Denies et al. (2015), the results of current study demonstrated major and language background play a significant role in determining language learners’ tendencies to communicate in and outside of the classrooms. In contrast, Kang (2014) argued that if language teachers focus on their teaching procedure and try to employ effective teaching strategies they can overlook language learners’ background in their teaching process.

The present study also investigated the impact of age on language learners’ WTC. The results discovered that there was a significant difference among the three groups with different age range. The results showed that those who were under 21 had a better performance than those that were under 18. Teachers always pay attention to this issue and try to recognize and differentiate the learners’ age in the classroom and to design and present the lesson in such a way that all students, despite their different abilities, achieve the maximum possible progress (Nematizadeh and Wood, 2019). According to the framework of the quality in education, the main axis of distinction between students is to observe the difference in their ability to learn (Lu and Hsu, 2008). Although this difference is the most important factor in the classroom, but in order to achieve a broader perspective on the issue of differentiation between students, it is better to look at other areas of difference, such as age, perceptual maturity, emotional status, gender, religion or racial group, and educational and family background. All of these factors can affect learners’ ability, their performance in the classroom, and their WTC (Joe et al., 2017). Just as teachers take for granted the differences in human faces, they must take for granted the difference in the abilities and needs of students. A knowledgeable teacher is a teacher who is familiar with the various abilities and inadequacies of their students and arranges their lessons in such a way that they develop in groups or individually according to their abilities and capabilities (Dewaele and Dewaele, 2018).

Language proficiency and its relation with WTC was another factor that was investigated in the current study. The results demonstrated that there were no statistically significant differences among language learners with different proficiency levels. These findings demonstrated that language learners could have difficulty in communication in every proficiency level. These findings confirmed what Fernández-García and Fonseca-Mora (2022) stated. They also found that while there is no difference between learners in different proficiency levels and their tendency to communicate. In contrast, the results of this study put the results of Zare et al. (2020) who found a statistically significant difference in learners’ WTC across proficiency levels under question. The results of the current study demonstrated that language learners in every proficiency level could have a high tendency to communicate or vice versa.

## Conclusion

Effective communication is one of the main pillars of social life. For many of us, communicating effectively requires learning some important skills. Learning these skills can deepen your relationship with others. This relationship also builds more trust and respect. Improve group work, problem-solving, and improve your overall social and emotional health. The present study investigated Chinese language learners’ WTC in terms of their gender, age, major, and proficiency level. The findings of the study demonstrated that the three factors play a crucial and considerable function in determining language learners’ WTC. However, the study found that learners in different proficiency levels have various levels of the tendency to communicate. In other words, WTC could be a problem for teachers at every language level.

Language learners, foreign language teachers, and anyone involved in teaching or learning a foreign language can benefit from the findings of the present study to some extent. Learners need to be fully aware of the major role they play in their tortuous language-learning path. They need to know that by using the power of emotions and feelings, having passion and motivation, being actively involved, and taking full responsibility for their own learning; almost nothing will stop them from achieving their goals. Foreign language teachers should consider the factors that motivate and motivate learners and do not use traditional methods as in the past. They should also consider personal and environmental factors in their training. They can take into account the age and gender differences of students and by using activities and exercises that improve and enhance the level of emotional intelligence, independent functioning, self-centeredness, and having enthusiastic participation in discussions and conversations. These exercises subsequently lead to an increase in the level of students’ WTC. For example, give more freedom of action to learners and less control over them, providing them with opportunities and situations in which people feel safe and secure and lead to more speech and interaction. Awareness of the importance of emotions and feelings in the success and advancement of goals, or by skillfully and unconsciously using strategies to learn activities that lead to increased emotional intelligence, freedom in decision-making, and self-confidence. Curriculum designers in the field of language learning as creators of the learning environment should pay special attention to psychological factors and individual differences, especially emotional intelligence, learner independence, self-regulation and WTC. Individual differences are vital factors in teaching and learning a foreign language, and many researchers and scholars have paid special attention to these factors in learning and teaching English as a foreign language.

Given the limitations as well as the lack of studies in this field, we make suggestions for future research. The same research can be done to evaluate the effect of emotional intelligence, self-regulation, and other skills such as listening, speaking, and writing skills. The learners’ proficiency level was indicated in the form of self-reported data, future studies can use more reliable procedure to check learners’ proficiency. Many individual differences such as self-esteem, self-efficacy beliefs, creativity, and critical thinking have not yet been studied in this area. In future studies, the relationship and effect of other motivational variables such as interest and the effect of different tasks and educational methods such as using the reverse educational method on motivation and WTC can be examined. In addition, qualitative methods such as observation and interviews, or longitudinal research can provide more comprehensible results.

## Data Availability Statement

The original contributions presented in the study are included in the article/supplementary material, further inquiries can be directed to the corresponding author.

## Ethics Statement

The studies involving human participants were reviewed and approved by Ethics Committee of Anhui University of Chinese Medicine. The patients/participants provided their written informed consent to participate in this study.

## Author Contributions

Both authors listed have made a substantial, direct, and intellectual contribution to the work, and approved it for publication.

## Conflict of Interest

The authors declare that the research was conducted in the absence of any commercial or financial relationships that could be construed as a potential conflict of interest.

## Publisher’s Note

All claims expressed in this article are solely those of the authors and do not necessarily represent those of their affiliated organizations, or those of the publisher, the editors and the reviewers. Any product that may be evaluated in this article, or claim that may be made by its manufacturer, is not guaranteed or endorsed by the publisher.
